# An infrared, Raman, and X-ray database of battery interphase components

**DOI:** 10.1038/s41597-024-04236-6

**Published:** 2025-01-08

**Authors:** Lukas Karapin-Springorum, Asia Sarycheva, Andrew Dopilka, Hyungyeon Cha, Muhammad Ihsan-Ul-Haq, Jonathan M. Larson, Robert Kostecki

**Affiliations:** 1 Energy Storage & Distributed Resources Division, Lawrence Berkeley National Laboratory, Berkeley, California 94720 USA; 2 Physics and Astronomy Department, Pomona College, Claremont, California 91711 USA; 3 Ulsan Advanced Energy Technology R&D Center, Korea Institute of Energy Research (KIER), Nam-gu Ulsan, 44776 Republic of Korea; 4 Department of Chemistry and Biochemistry, Baylor University, Waco, Texas 76798 USA

**Keywords:** Batteries, Batteries, Infrared spectroscopy, Raman spectroscopy, Energy

## Abstract

Further improvements to lithium-ion and emerging battery technologies can be enabled by an improved understanding of the chemistry and working mechanisms of interphases that form at electrochemically active battery interfaces. However, it is difficult to collect and interpret spectra of interphases for several reasons, including the presence of a variety of compounds. To address this challenge, we herein present a vibrational spectroscopy and X-ray diffraction data library of ten compounds that have been identified as interphase constituents in lithium-ion or emerging battery chemistries. The data library includes attenuated total reflectance Fourier transform infrared spectroscopy, Raman spectroscopy, and X-ray diffraction data, collected in inert atmospheres provided by custom sample chambers. The data library presented in this work (and online repository) simplifies access to reference data that is otherwise either diffusely spread throughout the literature or non-existent, and provides energy storage researchers streamlined access to vital interphase-relevant data that can accelerate battery research efforts.

## Background & Summary

Energy storage technologies play a large and growing role in humans’ lives and will continue to do so for the foreseeable future. Currently, rechargeable lithium-ion batteries (LIBs) have requisite energy and power densities for many applications as well as adequate cycle and calendar life at reasonable cost relative to other energy storage technologies^1,2^. As a result, LIBs (with various cathode materials) are used extensively in consumer electronics^3^, deployed in electric vehicles^4^, and integrated into electrical grids to store energy from intermittent renewable sources^1,5,6^. Innovations that increase battery safety and recyclability, reduce cost, mitigate supply chain concerns, and optimize the performance of LIBs could enable additional applications (e.g. long duration energy storage, short-haul aviation).

From a basic science perspective, many key performance characteristics of LIBs are enabled by the natural formation of interphases at electrochemically active electrode/electrolyte interfaces. The solid-electrolyte interphase (SEI) that forms at the anode/electrolyte interface is perhaps the most well-known, but the interphase that forms at the cathode/electrolyte interface – known as the cathode-electrolyte interphase (CEI) – is also relevant, especially when attempting to expand device energy density by increasing the upper cutoff voltage^7^. Ideally, interphases should regulate mass and charge transfer across the interface and passivate the electrode surface^8^. A critically important example is the SEI that forms at the interface between liquid organic electrolyte and graphite anode in modern LIBs. This SEI is known to conduct lithium ions – enabling ion intercalation and deintercalation during charging and discharging – but resist electrons, limiting undesirable side reactions with the electrolyte^9–11^. Moreover, and fortunately, this SEI naturally grows during initial cycling and is quite stable^8^. Because there is sometimes overlap between the chemical constituents of SEIs and CEIs depending on battery device chemistry and upper voltage cutoff^12^, we adopt the neutral/inclusive terminology of electrode-electrolyte interphase (EEI) in this work.

It is widely believed that the performance and lifetime of state-of-the-art LIBs and next-generation lithium batteries (e.g. those that utilize the conversion or plating of lithium or novel electrochemical intercalation chemistries) can be substantially improved through targeted engineering efforts informed by an enhanced understanding of the basic structure, chemistry, and working mechanisms of EEIs^13–16^. However, it is difficult to develop an understanding of fundamental structure-function relationships in EEIs in part because they are reactive, chemically heterogeneous, difficult to isolate and sense (being extremely thin, on the order of tens of nanometers thick), and because they are buried between dissimilar materials (e.g. electrode and electrolyte)^14^. Further, the composition of EEIs can be inconsistent between cells depending on manufacturing procedures, cycling conditions, and other physical circumstances which are not always standardized^9,11,14^.

Fourier transform infrared spectroscopy (FTIR) and Raman spectroscopy are commonly used to investigate EEI chemistry and structure while X-ray diffraction (XRD) can characterize the crystallinity of EEIs, which influences their ionic conductivity^11,14^. However, because these approaches are typically bulk scale characterization approaches, it is often difficult to interpret the data generated because both the numerous chemical components of the EEI, as well as other parts of the electrochemical cell (like active electrode materials and current collectors) may make contributions to the collected signal. Also, interactions between the many closely packed EEI nanograins of differing chemistry may skew spectra away from bulk counterparts. By various estimates, the anodic EEI in LIBs has more than ten unique chemical constituents^14,17^. In order to properly interpret the resulting complex spectra, reference measurements of individual candidate compounds are often used to identify contributions of individual compounds. Beyond being helpful for the bulk scale techniques, this approach to data interpretation is also extremely helpful in interpreting data from emerging nanoscale vibrational spectroscopy techniques, like infrared nanospectroscopy (nano-FTIR)^18,19^, that has recently been used to study electrochemical and battery EEIs^20–24^.

Although some spectral databases exist to assist in this, access to these can be prohibitively expensive or the databases are incomplete. Alternatively, researchers can collect reference spectra themselves or look to the literature. However, the former is time consuming and possibly costly, and the latter can be challenging for several reasons. First, published measurements of oxygen- and water-reactive compounds may include contributions from unwanted reaction products that can obscure features of interest. Second, the desired data is commonly difficult to find, being included in articles unrelated to EEI characterization, published in supplemental information sections, or plotted with other data. Finally, even if appropriate data is found in the literature, it is rarely available in a digitized form for streamlined use.

To reduce the practical challenges faced by energy storage researchers in the identification of EEI constituents, we present in this work, with corresponding online data library^25^, measurements of unreacted EEI components relevant to current and emerging LIB technologies, using attenuated total reflectance FTIR (ATR-FTIR), Raman, and XRD characterization instruments^25^. These compounds are lithium acetate, lithium carbonate, ^6^lithium fluoride, ^7^lithium fluoride, lithium hydride, lithium hexafluorophosphate, lithium oxide, manganese(II) fluoride, nickel(II) fluoride, and polyethylene oxide (PEO). The online data library^25^ contains both the final and raw data, as well as the fits that were subtracted from the raw data during data processing. Our results both confirm and expand upon those already in the literature and create the foundation for further efforts to generate a comprehensive database of reference measurements relevant to battery research. This work, and connected data library, will aid researchers in their efforts to more efficiently identify, or exclude, potential EEI components when analysing complex spectra and thus facilitate basic research of electrochemical interfaces of vital importance to battery materials science.

## Methods

The three characterization methods used – ATR-FTIR, Raman, and XRD – are detailed in the following subsections. Additionally, the protocols employed to ensure chemical compounds remained in an inert environment during storage, transfer, and characterization are briefly summarized and fully validated in the Technical Validation section. Prior to any characterization, all pristine compounds were stored in an argon glovebox with base oxygen and water concentrations of ~0.1 ppm and ~0.5 ppm, respectively. The sources and purities of the studied chemicals are provided in Table 1.

**Table 1:** Source and purity of compounds used for measurements presented in this paper.

Chemical Name	Chemical Formula	Purity	Supplier^d^	Product No.	Grain Size^a^
Lithium acetate	CH_3_COOLi	99.9%	SA	920320	60 um
Lithium carbonate	Li_2_CO_3_	99.999%	SA	752843	60 um
^6^Lithium fluoride	^6^LiF	99%^b^	SA	601411	60 um
^7^Lithium fluoride	^7^LiF	99.99%^c^	SA	449903	60 um
Lithium hydride	LiH	95%	SA	201049	700 um
Lithium hexafluoro-phosphate	LiPF_6_	98%	TF	011529.03	Unknown
Lithium oxide	Li_2_O	99.5%	TF^e^	041832.09	Unknown
Manganese(II) fluoride	MnF_2_	99%	TF	041832.09	150 um
Nickel(II) fluoride	NiF_2_	97%	TF	013067.09	Unknown
Polyethylene oxide	H(OCH_2_CH_2_)_n_OH	100%	TF	043678.14	Unknown

(a) Approximate value from the relevant supplier where available

(b) Advertised 95 atom percent ^6^Li, with total compound purity of 99%

(c) Advertised average molecular weight of 25.94 implies 92.6 atom percent ^7^Li, with total compound purity of 99.99%

(d) SA: Sigma Aldrich, TF: Thermo Fisher

(e) FTIR data taken using powder from American Elements (99.5%, LI-OX-025M-P)

### ATR-FTIR spectroscopy

ATR-FTIR spectra were collected from 370 to 4000 cm^−1^ at a spectral resolution of 2 cm^−1^ using a Shimadzu IRTracer-100 instrument with an IRIS single reflection diamond accessory. Herein, we generally report data in the mid-IR range, with a low-energy cutoff of about 500 cm^−1^. This approach was taken because the mid-IR range is accessible to experimentalists and because we use some data below 500 cm^−1^ to aid in the generation of a baseline for subtraction (see Data Processing for further details). We encourage those particularly interested in data around and below ca. 450 cm^−1^ to consult the raw data available in the online data library^25^.

The ATR-FTIR instrument was housed in a nitrogen-filled glovebox with an oxygen concentration below 20 ppm. We note that to the best of our knowledge, none of the compounds in this study react with nitrogen at room temperature, the main offenders are oxygen and water, whose concentrations were analogous to levels in an Ar glovebox. Compounds were transferred into the ATR-FTIR enclosure in sealed vials and then immediately placed on a clean diamond crystal for the ATR-FTIR measurement. This transfer approach was effective at minimizing unwanted reactions as described in detail in the Technical Validation section below. Most data presented here is an average of 512 individual spectra (CH_3_COOLi, Li_2_CO_3_, ^7^LiF, ^6^LiF, Li_2_O, PEO) which was used to maximize the signal to noise ratio, while only 50 spectra were accumulated for some of the more reactive compounds (LiH, LiPF_6_, MnF_2_, NiF_2_) to minimize acquisition time and thereby reduce the likelihood of undesired reactions with trace amounts of oxygen or water.

### Raman spectroscopy

**Fig. 1:**
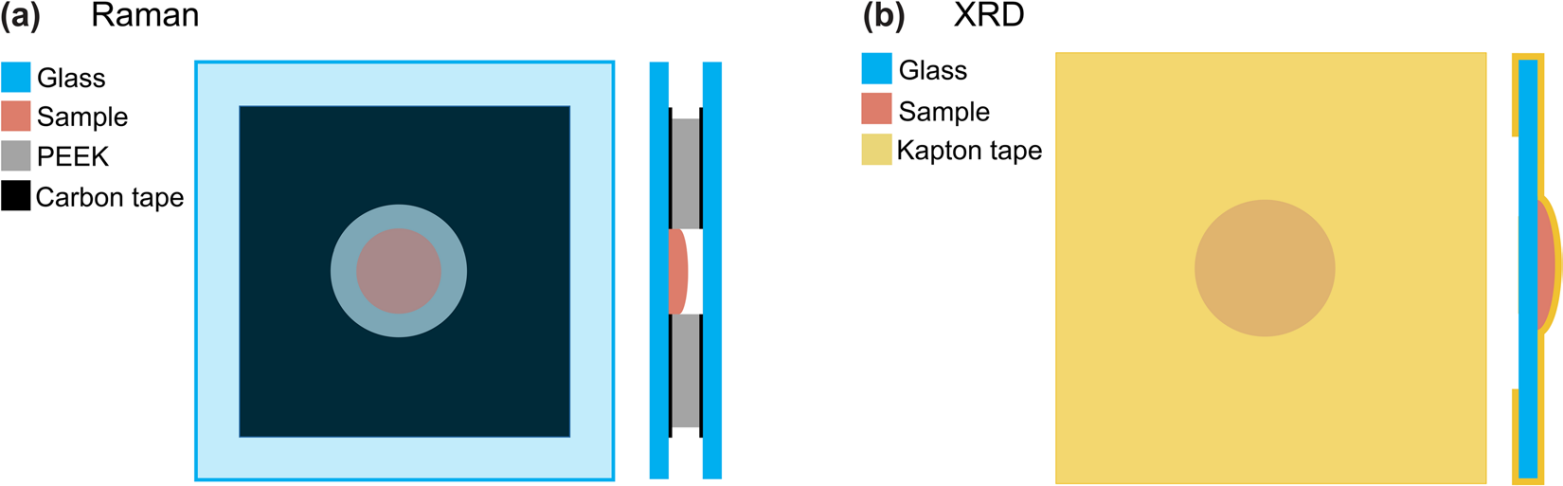
Designs of the sample chambers used to obtain (**a**) Raman and (**b**) XRD data.

Raman spectra were collected using a 2 cm-square and 5 mm thick custom-made polyether ketone (PEEK) sample chamber with an optical window (2.5 cm-square and 1 mm thick glass microscope slide). The chamber, which kept samples in an inert argon environment during measurement, is illustrated in Fig. 1a. Prior to cell assembly, PEEK pieces and glass slides were sonicated with acetone and then isopropyl alcohol and baked at 40°C for at least 4 hours before being transferred into an argon glovebox for assembly. After each sample chamber was assembled, it was isolated in a heat-sealed bag before being transferred to a Renishaw Qontor microscope where Raman spectroscopy was conducted. A 488 nm excitation laser was used at a power ranging from 1 to 10 mW to collect data over 25 acquisitions from 100 to 3200 cm^−1^. An additional measurement of the lithium oxide sample was performed on the same instrument using a 633 nm laser (see Table 9). Unwanted contributions to the Raman spectra from the glass optical window were avoided by focusing the laser on the surface of the sample inside the chamber.

### X-Ray diffraction

The sample chambers used for XRD measurements were similarly assembled in an argon glovebox. Small quantities of each compound were placed on clean 2.5 cm-square and 1 mm thick glass microscope slides (cleaned and dried using the method described above) and covered with several sealing overlayers of polyimide tape (Kapton, Ted Pella, silicone adhesive, 70 µm thick) before being heat-sealed in individual plastic bags. The sample chambers were then transferred to a Bruker Phaser D2 instrument (wavelength, λ = 1.54 Å) where X-ray diffraction patterns were collected over a 2θ range of 10 to 90 degrees using an acquisition time of 0.2 seconds per step and a step size of 0.02 degrees per step. All samples remained in their sealed bags until right before the measurement was started. Kapton tape is not perfectly air-tight but was a sufficient barrier to enable the acquisition of data, which was completed within the first 15 minutes after the sample chambers were brought into ambient air. The XRD patterns were collected through the tape, rather than through the glass slide, to prevent significant XRD contributions from the glass. The relatively smooth XRD background from the amorphous tape was removed via processing as described in the Data Processing subsection. The Technical Validation section provides evidence that this approach successfully minimized unwanted reactions.

### Data processing

The collected raw data was processed to isolate features of the spectra and diffraction patterns that can be used to identify the presence of these compounds in complex data collected from interphases. Unwanted instrumental and background contributions were also removed through this processing. All FTIR measurements of inorganic compounds – and some organic ones – contained strong and broad absorption features below 600 cm^−1^ which increased in intensity as the wavenumber decreased. These features were so broad that the decreasing intensity side of the feature (e.g. see Palik and Hunter’s data on LiF^26^) was not observed above the instrument’s low-energy detection limit of 370 cm^−1^. Because most researchers have detectors with similar limits (or even higher in energy), the FTIR data was processed to focus on the mid-IR regions (above ca. 500 cm^−1^) that are most commonly accessible experimentally. As a result, low-wavenumber features (below our reporting window) were fit as part of the baseline when defining baseline profiles to be subtracted from the raw data across the entire measured range, even though they are technically not part of the background. Raw, unsubtracted data spanning the entire measured range is available in the data library^25^. Panels 2a and 2b provide a representative baseline fitting of the raw data (which resulted in the removal of the downward-sloping part of the spectral feature that is observed below 600 cm^−1^) and the resulting subtracted data, respectively. Spikes in the Raman spectra attributable to cosmic ray excitation were removed immediately after collection and are not included in the raw data. Raw Raman spectra were processed through the subtraction of Gaussian and/or polynomial fits to eliminate background contributions that could have been caused by several phenomena including fluorescence, glass effects, and surface roughness. Because our instrument generated raw Raman data with unevenly spaced wavenumber values, we performed an interpolation was required to use a fast Fourier transform filter for data smoothing. Gaussian fits to determine peak positions from the data before and after interpolation confirmed that this transformation did not affect the location or shape of spectral features.

Gaussian fits were used to subtract the amorphous background that the Kapton overlayer generated in XRD measurements. This background between 10 and 30 degrees appeared in all measurements through Kapton tape (but not in control measurements of bare metal foils) and was at lower 2θ than the first diffraction peak of most compounds, allowing subtraction of a consistent background in the few patterns (like that of lithium acetate) where the first diffraction peak was found below 30 degrees. This approach was deemed suitable because the there were no consistent and unidentifiable peaks in the processed XRD patterns which would have indicated a residual contribution from the Kapton overlayer. A fast Fourier filter was applied to reduce high frequency noise (with care taken to avoid distorting spectral features) and small vertical offsets were used in some cases to align the high-2θ baseline near zero. All data was normalized to take on values from 0 to 1. To facilitate comparison with data collected on other instruments, d-spacing values (calculated using Braggs Law^27^ with λ = 1.54 Å) are included in the online repository in addition to a 2θ x-axis^25^.

### Mode assignment and notation

One of the contributions of this work is to synthesize existing knowledge about the vibrational modes and diffraction peaks of the studied compounds. These identifications are made on the data plotted in figures and in the accompanying tables. The notation used to identify peaks was standardized where possible and indicates the type of vibrational mode, the bond or functional group that generates the peak, and the symmetry of the vibration for FTIR and Raman spectra, and the Miller indices of the associated crystal plane for XRD patterns. Where this is not possible, the notation used in the literature is adopted and the reader is directed to references that provide additional details regarding the assignment of vibrational modes or crystal planes. Unless otherwise noted, Greek letters are used to describe vibrational modes according to the following scheme: *ω*, wagging; *δ*, bending; *ν*, stretching; *ρ*, rocking; *τ*, twisting. Subscripts “a” and “s” refer to asymmetric and symmetric modes, respectively. Similarly, the subscripts “i” and “o” refer to in-plane and out-of-plane modes, respectively. In the following subsections, we present the data for each compound organized by alphabetical order.

### Lithium Acetate - CH_3_COOLi

**Fig. 2:**
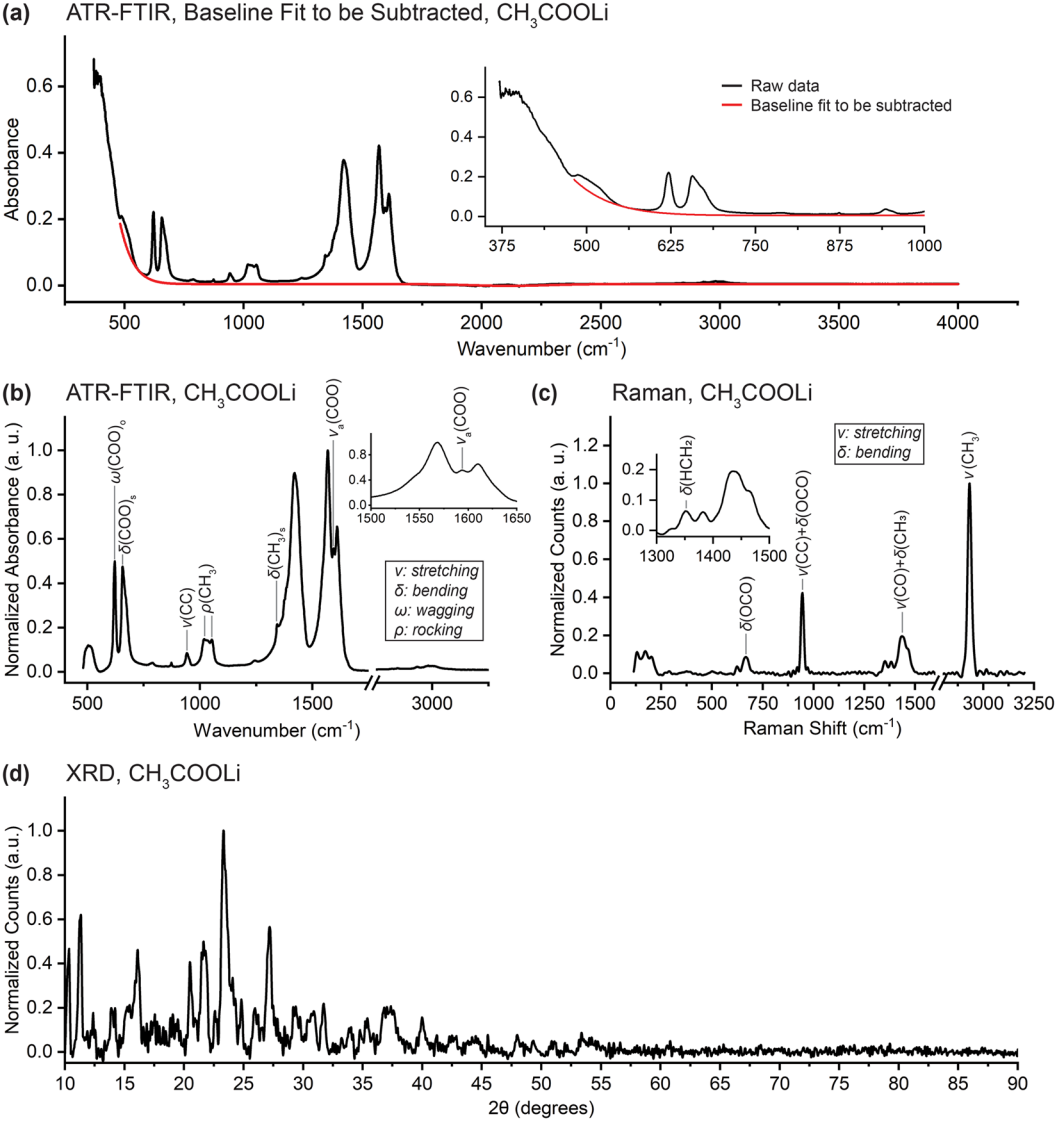
(**a**) Representative low-wavenumber baseline fit to be subtracted (red) from raw FTIR data for lithium acetate (CH_3_COOLi) (black) during processing with processed (**b**) ATR-FTIR, (**c**) Raman, and (**d**) XRD data. Peaks are identified from the literature: (**b**) Cadene^120^ and in (**c**) Sánchez-Carrera and Kozinsky^35^ and Ananthanarayanan^36^.

Lithium acetate has been identified as an EEI component in Li-O_2_ batteries^28^ and batteries with silicon anodes^29^. The baseline fit used to subtract out the low-wavenumber absorption feature is shown in Fig. 2a and is representative of those performed on the raw data of all of the inorganic compounds – and some of the inorganic ones – that we considered (see Data Processing for more details). The processed FTIR spectrum in Fig. 2b is in agreement with spectra reported elsewhere^30–34^. Assignments for most peaks are provided in the figure and in Table 2. The source of the peaks at 1421, 1568, and 1610 cm^−1^ remains unknown, although it has been proposed that they are generated by vibrational modes of the carboxylate group (COO^−^)^30^. Our data contains peaks at 503, 621, and 657 cm^−1^ which have occasionally been reported in the literature^32,33^. There is some disagreement in the literature as to the shape of the peak near 1600 cm^−1^ and our results agree best with those of Ross^34^ and Beyer *et al*.^31^. The Raman spectrum in Fig. 2c is in good agreement with the literature, where more detailed peak identifications (as well as empirical peak locations for lithium acetate dihydrate and the free acetate ion) can be found^35,36^. Peak identifications are made in the figure and in Table 3. The XRD pattern in Fig. 2d is in good agreement with that of the anhydrous polymorph obtained from the dehydration of CH_3_COOLi⋅2H_2_O^37^. Note that there is substantial variation within the literature as to the XRD pattern of lithium acetate, in part due to the dependency of the crystal structure on the specific hydrate (lithium acetate monohydrate and dihydrate as well as others) used as a precursor^37^ and also possibly due to differences in sample preparation procedures that resulted in varying degrees of isolation from water and oxygen.

**Table 2:** Peak assignments for the FTIR spectrum of lithium acetate.

This work (cm^−1^)	Literature^120^ (cm^−1^)	Assignment
621	621	*ω*(COO)_o_
657	660	*δ*(COO)_s_
942	943	*ν*(CC)
1020	1031	*ρ*(CH_3_)
1053	1055	*ρ*(CH_3_)
1343	1348	*δ*(CH_3_)_s_
1594	1595	*ν*_a_(COO)

**Table 3:** Peak assignments for the Raman spectrum of lithium acetate. Empirical locations of Raman peaks in lithium acetate dihydrate and the free acetate ion available in Ananthanarayanan^36^. (a) Assignment inferred from Ananthanarayanan^36^.

This work (cm^−1^) 488nm excitation	Literature^35^ (cm^−1^) [Calculated]	Assignment
667	—	^a^*δ*(OCO)
946	[942]	*ν*(CC) + *δ*(OCO)
1352	[1387]	*δ*(HCH_2_)
1435	[1440]	*ν*(CO) + *δ*(CH_3_)
2933	[2953]	*ν*(CH_3_)

### Lithium Carbonate, Li_2_CO_3_

**Fig. 3:**
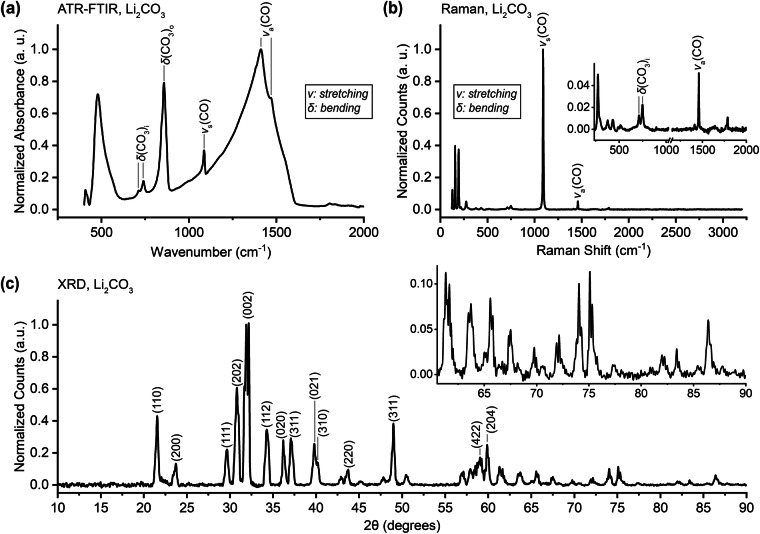
(**a**) ATR-FTIR, (**b**) Raman, and (**c**) XRD data for lithium carbonate (Li_2_CO_3_). Peaks are identified from the literature: (a) and (b) Pasierb *et al*.^43^ and (**c**) Chen *et al*.^51^.

Lithium carbonate forms in the anodic EEI of LIBs through the decomposition of ethylene carbonate^38^ and is also often found on the surface of lithium metal that is exposed to CO_2_^39^. The ATR-FTIR spectrum reported in Fig. 3a closely matches most of those previously reported^31,40–43^. Although some spectra are truncated well above 500 cm^−1^, Özer *et al*.^42^ and Pasierb *et al*.^43^ report a strong peak near 500 cm^−1^ and a weaker peak at slightly lower wavenumbers (both assigned to quasi-lattice vibrations^43^), which are similar to the peaks that we report at 477 and 408 cm^−1^. The Raman spectrum in Fig. 3b is in good agreement with the literature^43,44^. Further, the peaks that we report in the low-wavenumber region (at 128, 156, 194, and 274 cm^−1^) can be attributed to translational or rotational lattice vibrations of the carbonate ion (CO_3_^2-^)^45,46^. Peak identifications for FTIR and Raman-active vibrational modes are made in the figure and in Tables 4 and 5. Additional information about vibrational modes can be found in Brooker and Bates^47^, Brooker and Wang^48^, and Hase and Yoshida^45^. The XRD pattern in Fig. 3c confirms peak positions found in the literature and is consistent with a monoclinic crystal structure^49–51^. We report relative peak intensities that differ somewhat from previously reported values, although these values in the literature are generally not consistent with each other. Particular disagreement exists over the smaller peaks between 55 and 60 degrees, whose positions and relative intensities have not been agreed upon in the literature and which have been assigned to different crystal planes^49,51^. Notation is adopted from Chen *et al*.^51^ for consistency with the other peak assignments.

**Table 4:** Peak assignments for the FTIR spectrum of lithium carbonate.

This work (cm^−1^)	Literature^43^ (cm^−1^)	Assignment
713	712	*δ*(CO_3_)_i_
740	748	*δ*(CO_3_)_i_
857	863	*δ*(CO_3_)_o_
1087	1088	*ν*_s_(CO)
1412	1437	*ν*_a_(CO)
1468	1503	*ν*_a_(CO)

**Table 5:** Peak assignments for the Raman spectrum of lithium carbonate.

This work (cm^−1^) 488 nm excitation	Literature^43^ (cm^-1^) 1064 nm excitation	Assignment
710	712	*δ*(CO_3_)_i_
748	748	*δ*(CO_3_)_i_
1090	1088	*ν*_s_(CO)
1459	1458	*ν*_a_(CO)

### Lithium Fluoride - ^7^LiF & ^6^LiF

**Fig. 4:**
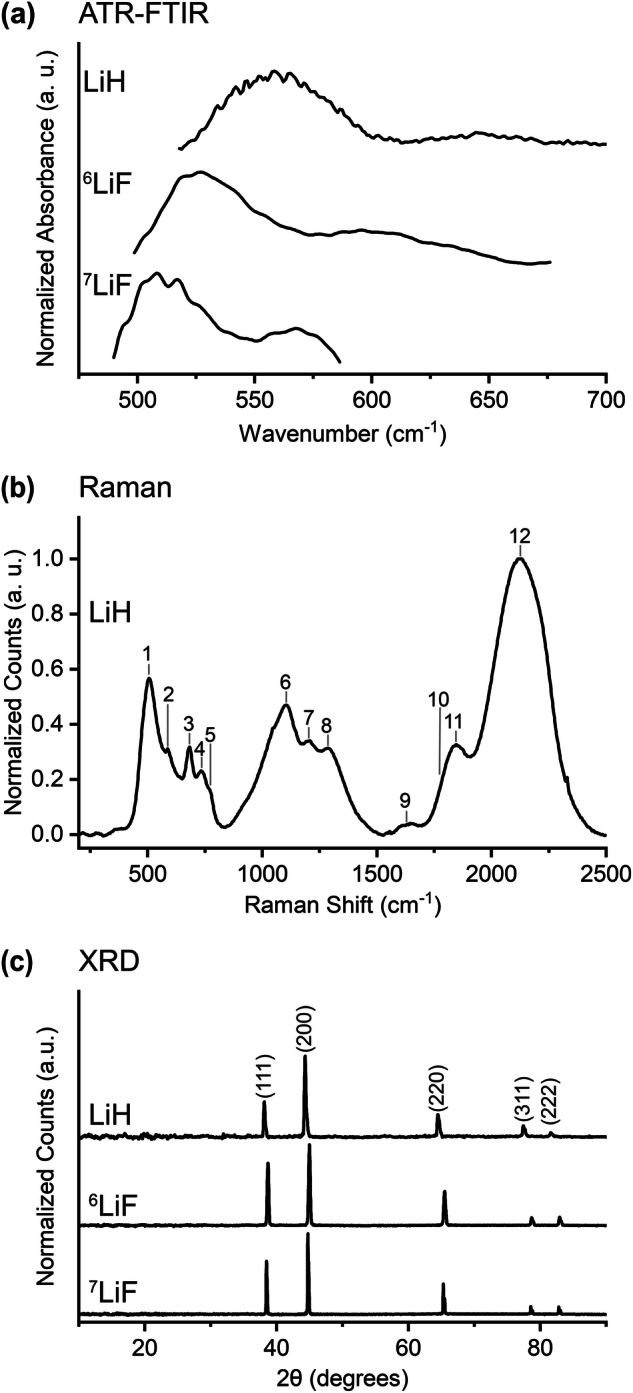
(**a**) ATR-FTIR, (b) Raman, and (c) XRD data for lithium fluorides (^7^LiF and ^6^LiF) and lithium hydride (LiH). Numeric labels in panel (**b**) correspond to linear combinations of optical and acoustic modes in the LiH crystal that are described in Tyutyunnik and Tyutyunnik^66^. Peak identifications in panel (**c**) apply to all three XRD patterns and are made from the following sources: LiH peak locations from Weber *et al*.^68^, ^6^LiF peak locations from Carturan *et al*.^123^, and ^7^LiF peak locations from Zhang *et al*.^59^ and Paterson^61^.

Lithium fluoride is a primary component of the initial EEI in LIBs and is produced when lithium hexafluorophosphate is reduced during a reaction with ethylene carbonate^38,52^. Both ^7^LiF and ^6^LiF are naturally occurring to some extent and their vibrational spectra are expected to be influenced by the differing atomic masses of lithium^53,54^. The ATR-FTIR spectra in Fig. 4a are similar to each other in that they have two broad features in the low wavenumber region (which we isolated from the large downward-sloping feature below 600 cm^−1^, as discussed in Data Processing). However, the peaks of the lighter ^6^LiF, at 527 and 595 cm^−1^, are shifted approximately higher than those of ^7^LiF (at 508 and 568 cm^−1^), in accordance with well-established theory about the effect of isotope masses on the frequency of vibrational modes^53^. These features contrast with previously reported features for LiF which have included a sharp peak near 800 cm^−1^ and the features of a spectrum collected of LiF in a high-temperature argon matrix^55,56^. A recent study of the nano-FTIR spectra of LiF describes how surface phonons significantly influence LiF spectra in certain circumstances^57^. While Raman data was collected, we do not plot it here because high fluorescence overwhelmed features of interest. The raw data is made available in the data library. As seen in Fig. 4c, both LiF samples produced XRD patterns that are consistent with their cubic structure and with patterns reported in the literature^58–60^. Note that data is often reported only through 80 degrees, excluding the peak at 83 degrees assigned to the 222 crystal plane^61^. We find that the diffraction peaks of ^6^LiF are shifted slightly to higher 2θ than those of ^7^LiF, which is qualitatively consistent with expected isotope effects^62^. Diffraction peaks for ^7^LiF around 79 and 83 degrees are assigned to the 311 and 222 crystal planes in the literature, and we extend these assignments to ^6^LiF on the basis of their shared cubic crystal structure and very similar unit cell size.

### Lithium Hydride – LiH

Lithium hydride is a common component of the EEI in lithium-anode batteries and has been identified in LIBs in the form of dendrites composed primarily of LiH that are associated with substantial capacity fading^63^. Importantly, LiH is sometimes misidentified as LiF due to similarities in their chemical properties, which will hopefully be reduced by the availability of the data presented here^64^. The ATR-FTIR spectrum in Fig. 4a contains peaks at 558 and 646 cm^−1^ which indicates the expected shift to higher wavenumber relative to the heavier lithium fluoride^53^. The main peak aligns with that in a previously reported spectrum, although we may be the first to report the weak secondary peak at 648 cm^−1^^ 65^. The Raman spectrum in Fig. 4b is similar to an existing spectrum and confirms that the sample has not reacted with oxygen or water to form Li_2_O or LiOH^66,67^. Peak assignments are adopted from Tyutyunnik and Tyutyunnik^66^ and the peak label “10” indicates the location where they find a modest peak that we do not observe in our data. Raman peak locations and assignments are also provided in Table 6. As seen in Fig. 4c, the XRD pattern of lithium hydride is similar to that of lithium fluoride, which has a comparable unit cell size and also has a cubic crystal structure. This is consistent with the literature^65,68,69^. The absence of peaks at 33 and 56 degrees, which are attributed to Li_2_O, confirms that the LiH remained unreacted throughout the measurement process^68^.

**Table 6:** Peak assignments for the Raman spectrum of lithium hydride. Detailed peak identifications provided in the ref.^66^.

This work (cm^−1^) 488 nm excitation	Literature^66^ (cm^−1^) 514.5 nm excitation	Assignment
514	511	1
592	586	2
688	687	3
748	736	4
779	776	5
1094	1109	6
1221	1211	7
1305	1293	8
1649	1638	9
—	1736	10
1833	1835	11
2142	2113	12

### Lithium Hexafluorophosphate - LiPF_6_

**Fig. 5:**
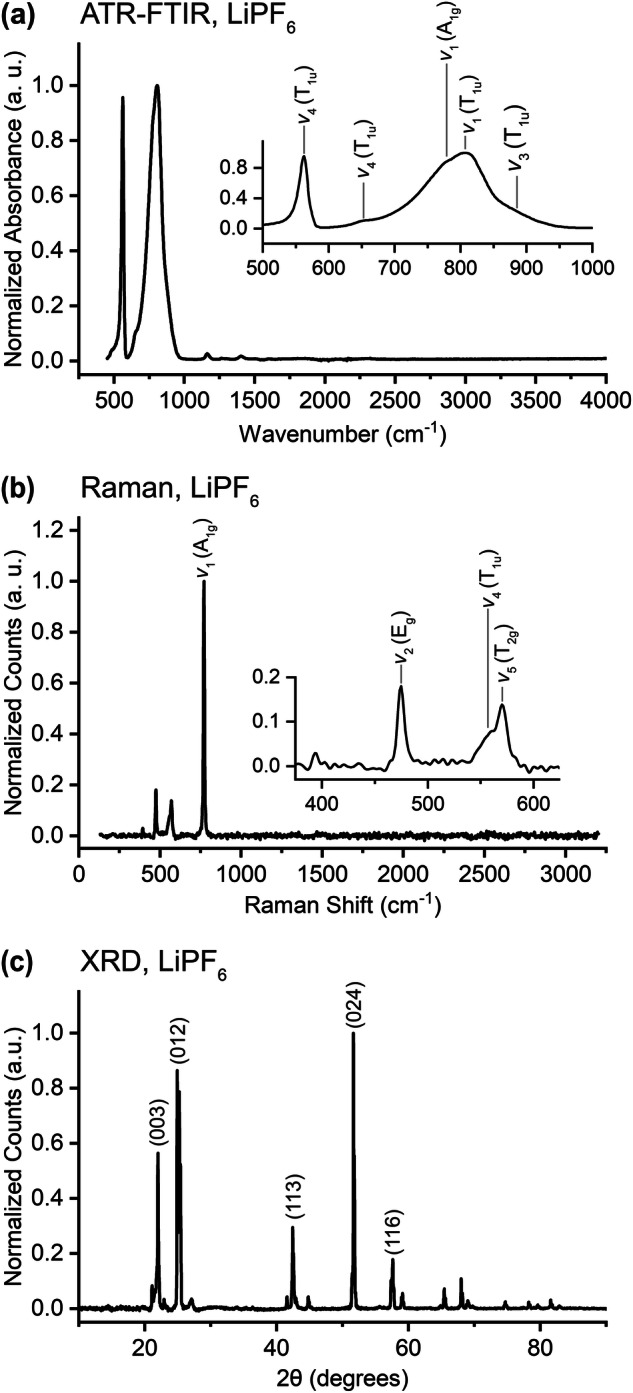
(**a**) ATR-FTIR, (**b**) Raman, and (**c**) XRD data for lithium hexafluorophosphate (LiPF_6_). Peaks are identified from the literature: (**a,b**) from Kock *et al*.^72^ and (**c**) from Masoud *et al*.^124^.

Lithium hexafluorophosphate is a common lithium source in the liquid electrolytes used in LIBs. As a result, residues of this salt are commonly found on and in the EEI, especially after the evaporation of the volatile components of the electrolyte^70^. The ATR-FTIR spectrum is provided in Fig. 5a. Peak assignments in the figure and Table 7 adopt the notation that is consistently used in the literature. Note that Pekarek *et al*.^71^ assign the peaks that they observe at 559 and 871 cm^−1^ to *δ*_s_(FPF) and *ν*_s_(PF), respectively, while we follow the assignment of Kock *et al*. for consistency. The Raman spectrum in Fig. 5b is in excellent agreement with previously reported values and peak identifications are provided in Table 8^72–74^. The *ν*_1_ vibrational mode at 771 cm^−1^ has been identified as arising from a P-F symmetric stretching mode^75^. It is worth noting that the normal modes of vibration of the PF_6_^-^ ion are dependent on the coordination of the ion and so can influenced by the local chemistry of a given EEI^75^. Moreover, partial oxidation of the PF_6_^-^ ion (which can occur upon electrochemical cycling) can also lead to changes in the position of the primary peaks^72^. Presented in Fig. 5c, the peak locations in the XRD pattern are in good agreement with the literature^72,74,76^. Further, we find relative peak intensities that fall in the wide range of results reported by others, with exception of a much stronger peak at 52 degrees than is reported elsewhere. There is some disagreement in the literature as to the crystal structure of LiPF_6_; Liu *et al*.^76^ suggest a hexagonal crystal structure and Kock *et al*.^72^ use Raman data to propose a cubic crystal structure like that of NaPF_6_ and KPF_6_.

**Table 7:** Peak assignments for the FTIR spectrum of lithium hexafluorophosphate. Notation adopted from Kock *et al*.^72^.

This work (cm^−1^)	Literature^72^ (cm^−1^)	Assignment
561	560	*ν*_4_(T_1u_)
650	646	*ν*_4_(T_1u_)
778	775	*ν*_1_(A_1g_)
805	798	*ν*_1_(T_1u_)
887	869	*ν*_3_(T_1u_)

**Table 8:** Peak assignments for the Raman spectrum of lithium hexafluorophosphate. Notation adopted from Kock *et al*.^72^.

This work (cm^−1^) 488 nm excitation	Literature^72^ (cm^−1^) 1064 nm excitation	Assignment
475	475	*ν*_2_(E_g_)
561	560	*ν*_4_(T_1u_)
571	571	*ν*_5_(T_2g_)
771	771	*ν*_1_(A_1g_)

### Lithium Oxide - Li_2_O

**Fig. 6:**
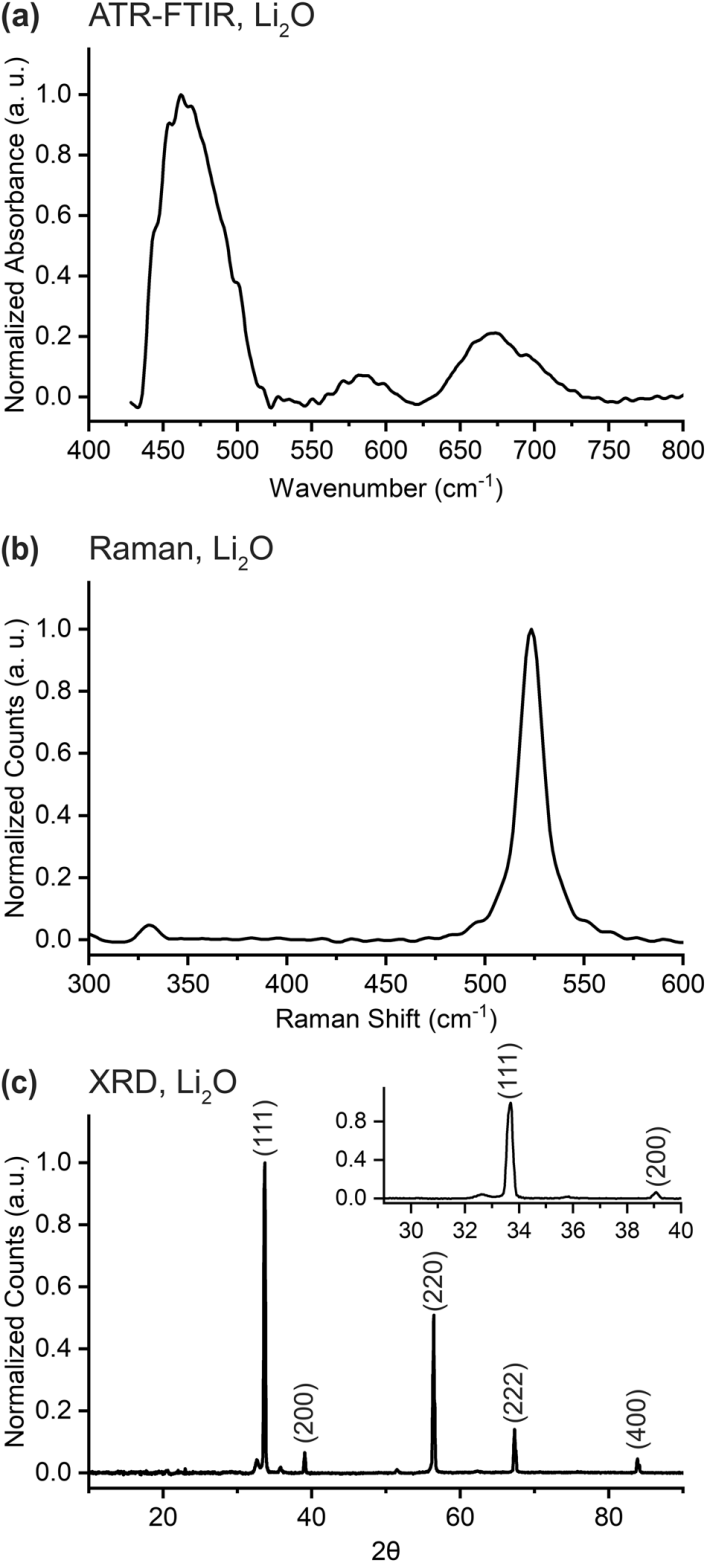
(**a**) FTIR, (**b**) Raman, and (**c**) XRD data for lithium oxide (Li_2_O). Peaks in panel (**c**) are identified from the Materials Project^125^.

Lithium oxide is believed to arise in the EEI as through the decomposition of lithium ethylene dicarbonate^38^ and also forms on the surface of lithium metal when it reacts with oxygen^39^. It can be difficult to obtain pristine spectra of lithium oxide because it reacts readily with water and because it is typically sold with some existing impurity^77^. While absorption features were observed at higher wavenumber values, ATR-FTIR data of Li_2_O is presented here in Fig. 6a below 800 cm^−1^ to focus on Li_2_O-specific features, which are fairly consistent with sparce reports in the literature (e.g. in the supplemental section of Tian *et al*.^78^). Features that emerged above the broad and downward-sloping low wavenumber absorption profile/baseline are isolated in the final data shown here. To verify infrared spectral features originating strictly from Li_2_O vibrations, we collected several spectra of powders exposed to gaseous water or CO_2_ or heated at 400 °C (to dehydrate the sample) for for various amounts of time. Through this process, some features could be attributed to Li_2_CO_3_ (870, 1432, and 1499 cm^−1^)^78–80^ and LiOH (3523, 3676 cm^−1^)^81^. because they scaled with exposure to moisture and CO_2_ at room temperature. Further, one absorption feature (at ~3523 cm^−1^) was found to decrease in intensity with increasing dehydration by way of heating and was assigned to LiOH⋅H_2_O^82,83^ in accord with past reports, while the peak at 3676 cm^−1^ was assigned to the O-H stretching mode in LiOH. The choice of scale in Fig. 6a was informed by the fact that these “impurities” were all found above 800 cm^−1^.

The Raman spectrum of lithium oxide is provided in Fig. 6b. The primary peak from an Li-O vibration (in the *F*_2g_ symmetry group^84^) at 523 cm^−1^ is congruent with the range of peak positions, from 515 to 529 cm^−1^, reported in the literature^77,85^ (including the Supplemental Materials section of Gittleson *et al*.^86^) and precisely confirms the experimental result of Sánchez-Carrera and Kozinsky^35^ and Osaka and Shindo^84^. The variance in the previously reported peak locations may be due to differences in the excitation laser wavelength used, as seen in Table 9, although the peak location in our data remained unchanged when switching between 488 and 633 nm excitation lasers. The collected XRD pattern in Fig. 6b is in good agreement with data reported elsewhere and is consistent with a cubic crystal structure^87,88^.

**Table 9:** Energy level of the primary peak of lithium oxide’s Raman spectrum across studies.

Excitation Wavelength (nm)	Primary Peak Location (cm^−1^)	Reference
325	515	Sifuentes *et al*.^85^
488	523	This work
488	523	Osaka and Shindo^84^
514.5	523	Osaka and Shindo^84^
532	527	Weber *et al*.^77^
633	523	This work
785	529	Gittleson *et al*.^86^

### Manganese(II) Fluoride - MnF_2_

**Fig. 7:**
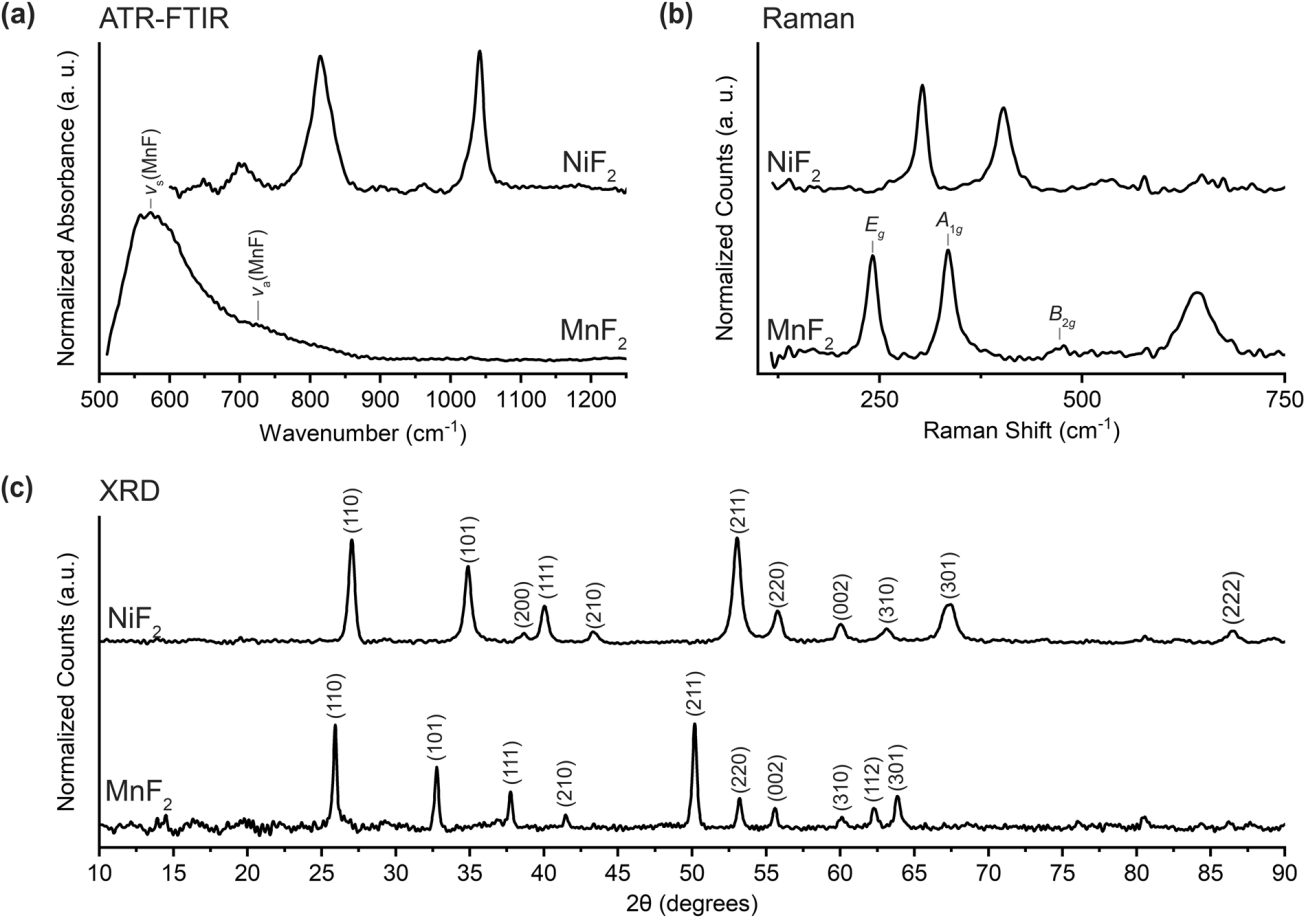
(**a**) ATR-FTIR, (**b**) Raman, and (**c**) XRD data for manganese(II) fluoride (MnF_2_) and nickel(II) fluoride (NiF_2_). Peaks are identified from the literature: (**a**) Scholz and Stösser^93^, (**b**) Stavrou *et al*.^94^, (**c**) Rui *et al*.^95^ for MnF_2_ and Jiao *et al*.^102^ for NiF_2_.

Manganese(II) fluoride is hypothesized to form in the EEI on manganese-containing metallic glass anodes following reactions with LiPF_6_ salt in the electrolyte^89^ as well as on other manganese-rich electrodes^90–92^. The ATR-FTIR spectrum presented in Fig. 7a contains peaks similar to those calculated and empirically reported by Scholz and Stösser^93^ as seen in Table 10. We find a peak at 575 cm^−1^ that may be generated by the symmetric stretching mode whose position they calculated but which they did not experimentally report^93^. The Raman spectrum in Fig. 7b agrees closely with previously reported values shown in Table 11^94^. In lieu of vibrational mode assignments, we provide the symmetry groups of the phonon modes that are believed to generate the peaks that we observe in the low wavenumber region. The peak at 641 cm^−1^ remains unassigned. Seen in Fig. 7c, the XRD data suggests a tetragonal structure and is in good agreement with published patterns^95–97^. While relative peak intensities are generally similar to those found in the literature, the peak at 50 degrees is somewhat stronger than reported elsewhere.

**Table 10:** Peak assignments for the FTIR spectrum of manganese(II) fluoride. Calculated frequencies are provided in square brackets in addition to empirically observed frequencies.

This work (cm^−1^)	Literature^93^ (cm^−1^)	Assignment
575	− [529-599]	ν_s_(MnF)
726	700 [673-768]	ν_a_(MnF)

**Table 11:** Peak assignments for the Raman spectrum of manganese(II) fluoride. Calculated frequencies are provided in square brackets in addition to empirically observed frequencies. Peak identifications refer to phonon modes.

This work (cm^−1^) 488 nm excitation	Literature^94^ (cm^−1^) 488 nm excitation	Assignment (Phonon Mode)
247	245 [233]	E_g_
341	340 [350]	A_1g_
476	457 [463]	B_2g_

### Nickel(II) Fluoride - NiF_2_

Nickel(II) fluoride is believed to form in the EEI on lithium nickel cobalt manganese oxide (NCM) cathodes in LIBs^98,99^. There is substantial disagreement in the literature as to the correct FTIR spectrum; our data, shown in Fig. 7a, has primary peaks at 817 and 1043 cm^−1^ and most closely resembles that of Tramšek *et al*.^100^. In addition to these primary peaks, Tramšek *et al*. report secondary peaks that are similar to those that we find at 651 and 706 cm^−1^. However, these are more prominent in our data because we perform a background subtraction to isolate these features (as described in Data Processing). Presented in Fig. 7b, our Raman spectrum shows peaks at 303 and 405 cm^−1^ in good agreement with Ullah *et al*.^101^. The XRD pattern in Fig. 7c closely matches those reported in the literature with respect to both peak locations and relative intensities (with the exception of the strong peak that we report at 53 degrees) and is consistent with the expected tetragonal structure^102–104^. The broad diffraction peak near 67 degrees has been suggested to be caused by the overlap of two adjacent peaks stemming from the 301 and 112 crystal planes, but our instrument does not have sufficient resolution for us to make this identification^103^.

### Polyethylene oxide - H(OCH_2_CH_2_)_n_OH

**Fig. 8:**
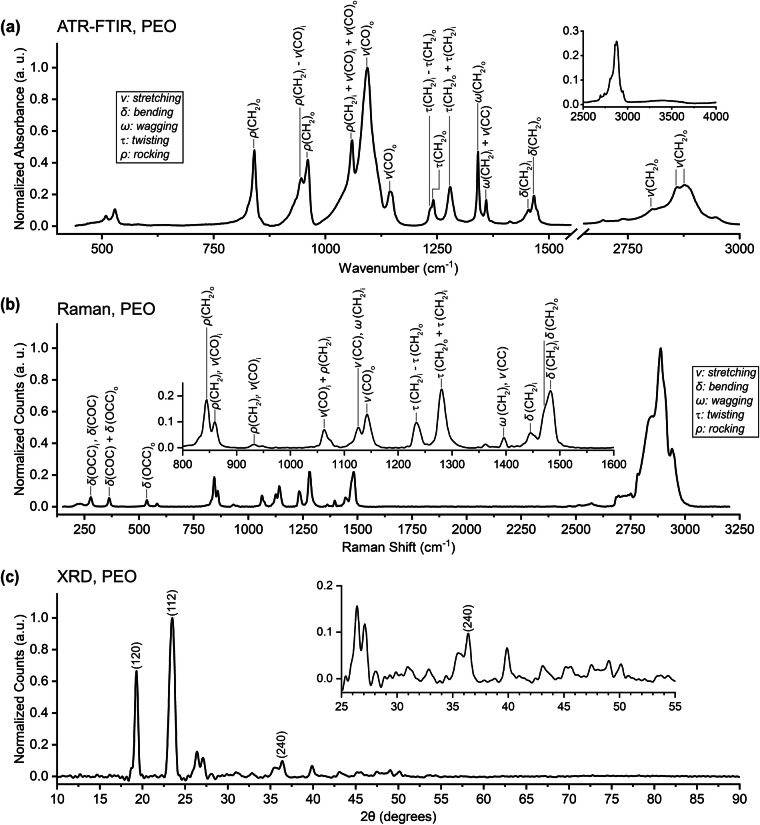
(**a**) ATR-FTIR, (**b**) Raman, and (**c**) XRD data for polyethylene oxide (H(OCH_2_CH_2_)_n_OH). Peaks are identified from the literature: (**a**) Matsui *et al*.^121^ and Ratna *et al*.^122^, (**b**) Matsui *et al*.^121^, and (**c**) Telfah *et al*.^126^ Phase relations of “+” and “-” are provided for coupled coordinates where available but are otherwise replaced with a comma.

Polyethylene oxide is a polymer that is used in solid polymer electrolytes and as a component in artificial EEIs for anode-free batteries^105,106^. PEO oligomers have also been identified in the EEI on silicon anodes where they are believed to be products of electrolyte reduction^107^. Seen in Fig. 8a, the FTIR spectrum that we present contains the low-wavenumber peaks at 509 and 530 cm^−1^ and the peak at 2876 cm^−1^ reported in some works^108–111^ but only faintly shows the broad feature near 3370 cm^−1^ found by some^108–110^ but not others^111,112^. We provide peak locations and assign them to vibrational modes in the figure and in Table 12; phase relations of “ + ” and “-” are provided for coupled coordinates where available but are otherwise replaced with a comma. Additional discussions of vibrational modes can be found in the literature^113^. The Raman spectrum presented in Fig. 8b is in good agreement with the existing literature as seen in Table 13^114–116^. The XRD pattern in Fig. 8c aligns well with those reported elsewhere^117–119^.

**Table 12:** Peak assignments for the FTIR spectrum of polyethylene oxide. Calculated frequencies in square brackets. Phase relations of “+” and “-” are provided for coupled coordinates where available but are otherwise replaced with a comma.

This work (cm^−1^)	Literature^121,122^ (cm^−1^)	Assignment
841	844 [856]	*ρ*(CH_2_)_o_
946	947 [924]	*ρ*(CH_2_)_i_ − *ν*(CO)_i_
961	958 [884]	*ρ*(CH_2_)_o_
1060	1060 [1033]	*ρ*(CH_2_)_i_ + *ν*(CO)_i_ + *ν*(CO)_o_
1094	1103 [1061]	*ν*(CO)_o_
1144	1147 [1161]	*ν*(CO)_o_*
1236	1234 [1250]	*τ*(CH_2_)_i_ − *τ*(CH_2_)_o_
1241	1240 [1280]	*τ*(CH_2_)_o_
1279	1278 [1282]	*τ*(CH_2_)_o_ + *τ*(CH_2_)_i_
1342	1342 [1386]	*ω*(CH_2_)_o_
1360	1358 [1354]	*ω*(CH_2_)_i_ + *ν*(CC)
1454	1448 [1470]	*δ*(CH_2_)_i_
1467	1466 [1474]	*δ*(CH_2_)_o_
2806	2750-3000	*ν*(CH_2_)_o_
2861	2750-3000	*ν*(CH_2_)_o_
2876	2750-3000	*ν*(CH_2_)_o_

**Table 13:** Peak assignments for the Raman spectrum of polyethylene oxide. Calculated frequencies in square brackets. Phase relations of “+” and “-” are provided for coupled coordinates where available but are otherwise replaced with a comma. (a) Alternative identification is made in Tadokoro *et al*.^113^.

This work (cm^−1^) 488 nm excitation	Literature^121^ (cm^−1^) 435.8 nm excitation	Assignment
279	274 [270]	*δ*(OCC)_i_, *δ*(COC)
363	359 [366]	*δ*(COC) + *δ*(OCC)_i_
536	531 [501]	*δ*(OCC)_o_^a^
844	844 [856]	*ρ*(CH_2_)_o_
860	859 [876]	*ρ*(CH_2_)_i_, *ν*(CO)_i_
933	930 [924]	*ρ*(CH_2_)_i_, *ν*(CO)_i_
1062	1066 [1093]	*ν*(CO)_i_ + *ρ*(CH_2_)_i_
1126	1130 [1138]	*ν*(CC), *ω*(CH_2_)_i_
1142	1147 [1161]	*ν*(CO)_o_^a^
1231	1237 [1250]	*τ*(CH_2_)_i_ − *τ*(CH_2_)_o_
1280	1283 [1282]	*τ*(CH_2_)_o_ + *τ*(CH_2_)_i_
1396	1398 [1381]	*ω*(CH_2_)_i_, *ν*(CC)
1446	1447 [1470]	*δ*(CH_2_)_i_
1470	1474 [1474]	*δ*(CH_2_)_o_
1480	1483 [1473]	*δ*(CH_2_)_i_

## Data Records

All data presented or discussed herein can be found in the Dryad data library associated with this work^25^. The data files are in a.xlsx format which can be opened using Excel (including the free Excel Viewer) and Google Sheets among other applications. These files can then be exported as a.csv or other formats as desired for use in data processing and plotting software. One Excel workbook file is provided for each characterization technique (ATR-FTIR, Raman, and XRD). Each sheet in the workbook contains data for one compound, whose identity is indicated by the chemical formula and written name. Raw and final data are provided, and the ATR-FTIR and XRD data files also each contain an additional sheet with all final data compiled with a common x-axis. Note that final Raman data for ^6^LiF and ^7^LiF are omitted as discussed in the Methods section, and that raw Raman data was interpolated into even x-axis spacing prior to the application of a Fourier filter. As a result, there are therefore different wavenumber axes for the raw and final Raman data; the axes are provided in each sheet and are visually offset by an empty column. An additional column in the XRD file contains d-spacing values calculated from the wavelength of the instrument (λ = 1.54 Å) using Bragg’s Law^27^. The.txt file in the repository folder contains all of the information provided in this section.

## Technical Validation

The procedures outlined in the Methods section were designed to ensure that we measured compounds in their unreacted state. We were successful in doing so, as confirmed through the comparison of our data to literature results of both reacted and pristine compounds of interest. Comparison of the ATR-FTIR spectrum of LiPF_6_ (shown in Fig. 5a) to the literature demonstrates that our measurement procedure effectively prevented degradation of the sample; the LiPF_6_ is demonstrably unreacted^72,74^. We were able to verify our approach through the LiPF_6_ data because there exists reference data for this compound in its pristine and reacted forms, which was not available for the other compounds studied in this work. The efficacy of our Raman measurement protocols is supported by the lithium hydride spectrum in Fig. 4b; the absence of peaks at 523 cm^−1^ (from Li_2_O) and between 250 and 350 cm^−1^ (attributed to LiOH)^67^ provides strong evidence that this highly oxygen- and water-sensitive compound did not degrade into its common reaction products. Further, the quality of the XRD procedures is confirmed by the lithium oxide data in Fig. 6c; the peaks generated by Li_2_O are easily identified and there are almost negligible contributions at ~33 degrees from the 101 plane of LiOH (which have been suggested to arise from impurities in the as-delivered powder^77^) and no detectable contributions at ~36 degrees from the 110 plane of LiOH⋅H_2_O. This is strong evidence that the sample was well protected from oxygen and had no detectable exposure to water^77^. Similarly, the absence of peaks at 33 and 56 degrees in the XRD pattern of LiH in Fig. 4c confirms the absence of LiOH contamination in the LiH^68^.

Our data also closely aligns with previously reported spectra and patterns (where these are available) for FTIR (lithium acetate^31,34^, lithium carbonate^42,43^, lithium hydride^65^, lithium hexafluorophosphate^72,74^, nickel(II) fluoride^100^, polyethylene oxide^112^), Raman (lithium acetate^35,36^, lithium carbonate^43,44^, lithium hydride^66^, lithium hexafluorophosphate^72–74^, lithium oxide^35,84^, manganese(II) fluoride^94^, nickel(II) fluoride^101^, polyethylene oxide^114–116^), and XRD (lithium acetate^37^, lithium carbonate^49–51^, ^6^lithium fluoride^60^, ^7^lithium fluoride^58,59^, lithium hydride^65,68,69^, lithium hexafluorophosphate^72,74,76^, lithium oxide^77,87,88^, manganese(II) fluoride^95–97^, nickel(II) fluoride^102–104^, polyethylene oxide^117–119^).

## Data Availability

No code was used in this study.
